# More human than human: a Turing test for photographed faces

**DOI:** 10.1186/s41235-019-0197-9

**Published:** 2019-11-21

**Authors:** Jet Gabrielle Sanders, Yoshiyuki Ueda, Sakiko Yoshikawa, Rob Jenkins

**Affiliations:** 1 Department of Psychology, University of York, York, UK; 2 Department of Psychology and Behavioural Science, London School of Economics and Political Sciences, London, UK; 3 Kokoro Research Center, Kyoto University, Kyoto, Japan

**Keywords:** Hyper-realistic face masks, 2AFC, Other-race effect, Turing test, Synthetic faces, Deliberate disguise, Silicone masks

## Abstract

**Background:**

Recent experimental work has shown that hyper-realistic face masks can pass for real faces during live viewing. However, live viewing embeds the perceptual task (mask detection) in a powerful social context that may influence respondents’ behaviour. To remove this social context, we assessed viewers’ ability to distinguish photos of hyper-realistic masks from photos of real faces in a computerised two-alternative forced choice (2AFC) procedure.

**Results:**

In experiment 1 (*N* = 120), we observed an error rate of 33% when viewing time was restricted to 500 ms. In experiment 2 (*N* = 120), we observed an error rate of 20% when viewing time was unlimited. In both experiments we saw a significant performance cost for other-race comparisons relative to own-race comparisons.

**Conclusions:**

We conclude that viewers could not reliably distinguish hyper-realistic face masks from real faces in photographic presentations. As well as its theoretical interest, failure to detect synthetic faces has important implications for security and crime prevention, which often rely on facial appearance and personal identity being related.

## Significance

Forensic identification often relies on comparison of facial images (photographs or video stills) by human viewers. There are now dozens of criminal cases in which perpetrators have used hyper-realistic face masks to transform their appearance (e.g. change in apparent age, sex, or race). Facial disguise is not a new problem, but the level of realism that is achievable with these masks does raise new questions. With conventional disguises (e.g. balaclava or domino mask), it is generally clear that captured images do not show the person’s actual appearance. With hyper-realistic face masks, the situation is very different. Beyond a certain level of realism, viewers might think that captured images show the wearer’s real face. An error of that type can set an investigation down the wrong path, as numerous recent cases have shown (e.g. searching for a suspect of the wrong race). All of these implications hinge on whether or not the masks are truly realistic. Here we address this question by developing a Turing Test for photographed faces.

## Background

Technologies often imitate natural objects, giving rise to artificial diamonds, artificial flowers, artificial fur, and countless other artefacts. How are we to judge the success of such imitations? In 1950, Alan Turing proposed an influential answer for the specific case of artificial intelligence: an imitation is successful when we cannot distinguish it from the real thing (Turing, 1950). In his original argument, Turing imagined a human evaluator engaged in natural language conversations with a real human and a computer designed to generate human-like responses. The evaluator would be informed that one of the two partners is a computer, and asked to determine which one. To focus the evaluation on quality of thought rather than quality of speech, the dialogue would be mediated by text only (e.g. keyboard and screen). If the evaluator cannot reliably distinguish the computer from the human, the computer is said to pass the test.

As a target of imitation, intelligent conversation is enormously complex. No current machine appears close to passing the Turing test. However, the logic of the test itself is straightforward, and provides a means for assessing the maturity of imitation technologies generally: given the imitation alongside the real thing, can an observer tell which is which?

**Fig. 1:**
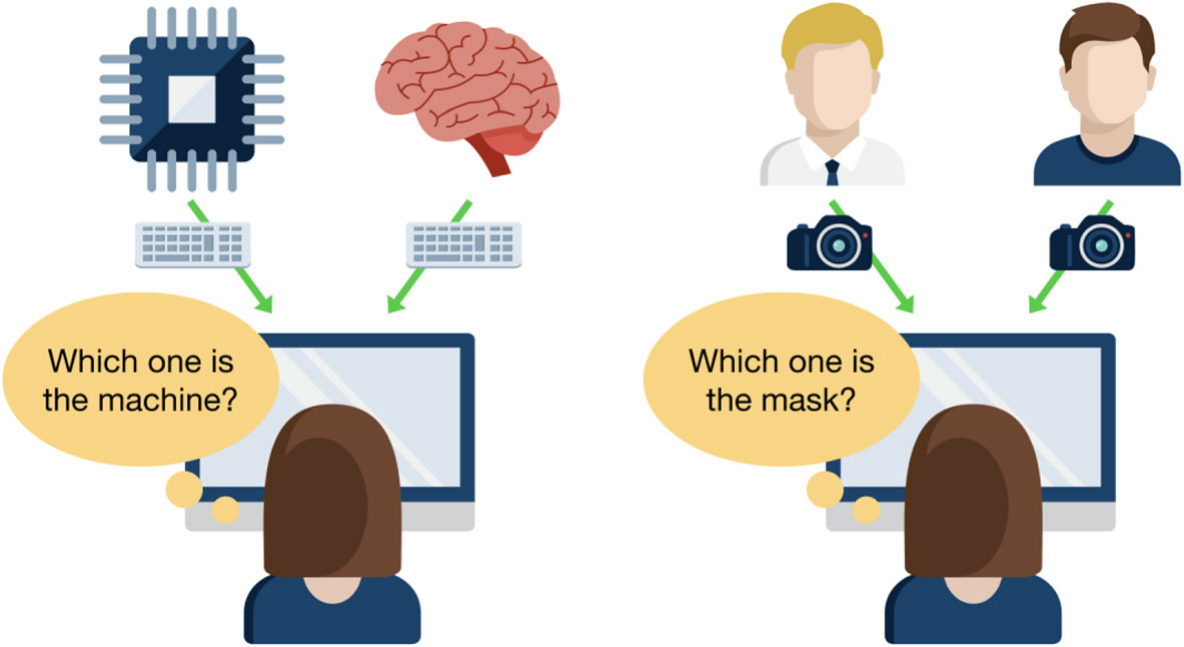
Schematic illustrating parallels between the standard Turing test (left) and a similar test for synthetic faces (right). In both cases, an evaluator is given the task of trying to determine which presentation is the genuine article and which is the imitation. The evaluator is limited to using a computer interface to make the determination

Here we bring this logic to bear on a much more tightly circumscribed imitation technology - artificial faces (see Fig. 1). The past decade has seen increasing interest in the realism of computer-generated faces (Holmes, Banks, & Farid, 2016; Nightingale, Wade, & Watson, 2017). Our concern is artificial face images of a very different kind, specifically, unretouched photos of artificial faces in the real world. Images in this category differ from digital images in at least two important ways. First, digitally generated or manipulated images are not snapshots of the physical environment. They only exist in print and on screen, and that limits the ways in which viewers can encounter them. Our focus is physical artefacts that exist in the real world and are caught on camera. Second, digital image manipulation has been a part of mainstream media for a generation. As such, the level of public understanding that images may be “photoshopped” is high. One consequence of this development is that photorealistic images carry less evidential weight than they once did - all images are suspect in this sense (see Kasra, Shen, & O’Brien, 2018). Since the real world cannot be photoshopped in the same way, physical artefacts are more protected from this slide in credibility.

**Fig. 2:**
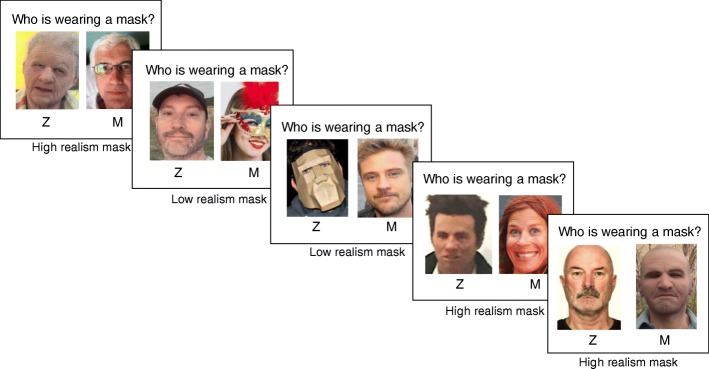
Example trials from the Caucasian image set. Each mask image was randomly paired with one real-face image from the set, independently set for each participant. Correct answers from left to right: Z, M, Z, Z, M. For source information, see Additional file 1

Artificial faces in the real world may not be intended to pass for genuine faces, even when they strive for realism in some respect. A marble bust might capture the proportions of a real face, but none of the movement; a robotic head might capture some facial movement, but remain disembodied. Hyper-realistic silicone masks differ from these examples in that they are worn by a real person, and so are seen in the context of a real body. Moreover, they are constructed from a flexible material, so they relay the wearer’s rigid and non-rigid head movements - at least at the gross scale (e.g. head turns; opening and closing of the mouth). These characteristics set hyper-realistic masks apart from other artificial faces, as they allow them to be fully embedded in natural social situations (see Fig. 2 for examples).

These natural social situations place unusual demands on imitation technologies, as humans tend to be especially attuned to social stimuli. Face perception offers abundant evidence of such tuning. For example, humans are predisposed to detect face-like patterns (Robertson, Jenkins, & Burton, 2017), and this tendency is present from early infancy (Morton & Johnson, 1991). Faces capture our attention (Langton, Law, Burton, & Schweinberger, 2008; Theeuwes & Van der Stigchel, 2006), and having captured attention, tend to retain it (Bindemann, Burton, Hooge, Jenkins, & De Haan, 2005). While viewing a face, we make inferences about the mind behind it, including emotional state from facial expression (Ekman & Friesen, 1971; Ueda & Yoshikawa, 2018; Young et al., 1997) and direction of attention from eye gaze (Baron-Cohen, Wheelwright, Hill, Raste, & Plumb, 2001; Friesen & Kingstone, 1998). We also use faces to identify individual people (Burton, Bruce, & Hancock, 1999; Burton, Jenkins, & Schweinberger, 2011), which can trigger retrieval of personal information from memory (Bruce & Young, 1986). All of these processes require high sensitivity to subtleties of facial appearance. There is even some evidence that these processes can become tuned to specific populations through social exposure. For example, children tend to be better at recognising young faces than old faces (and vice versa; Anastasi & Rhodes, 2005; Neil, Cappagli, Karaminis, Jenkins, & Pellicano, 2016); Japanese viewers tend to be better at recognising East Asian faces than Western faces (and vice versa; O’Toole, Deffenbacher, Valentin, & Abdi, 1994). Perhaps most relevant for the current study, discrimination between faces and non-face objects can be accomplished rapidly and accurately. Using saccadic reaction times, Crouzet, Kirchner, and Thorpe (2010) found that viewers could differentiate images of faces versus vehicles at 90% accuracy in under 150 milliseconds - significantly faster than discriminations that did not involve faces. The findings of Crouzet et al. (2010) were based on images from different categories. Nevertheless, they provide an interesting baseline against which to compare the more nuanced discriminations investigated here.

Taken together, these findings suggest that faces may be particularly difficult objects to imitate. Faces attract the glare of attention, and details of their appearance convey socially significant information. Even so, there is some evidence that hyper-realistic silicone masks can pass for real faces, at least in certain situations. In a previous study (Sanders et al., 2017), passers-by consistently failed to notice that a live confederate was wearing a hyper-realistic mask, and showed little evidence of having detected the mask covertly. Out of 160 participants in the critical condition, only two spontaneously reported the mask, and only a further three reported the mask following prompting. These low detection rates are consistent with the idea that hyper-realistic masks successfully imitate real faces. However, several aspects of the experimental procedure complicate this interpretation. For example, masks were not mentioned during the main phase of data collection, and participants had no reason to expect to see a mask. It is possible that participants might have detected the masks more often had they been expecting them. Moreover, responses were collected in a live social setting. It is possible that respondents were reluctant to inspect or to discuss the appearance of a person who was physically present (albeit out of earshot) - and especially reluctant to declare that person’s face to be artificial.

These matters of interpretation arise in part from our approach to testing, which prioritised ecological validity over experimental control. Here we adopt the complementary approach of two-alternative forced choice testing (2AFC), which strikes the opposite balance (see Bogacz, Brown, Moehlis, Holmes, & Cohen, 2006 for a review). The 2AFC method originated in psychophysical research (Fechner, 1860/1966), where it was developed to measure quantities such as perceptual acuity. Our application is closer in spirit to the Turing test, in that our main interest concerns the realism of artificial stimuli.

In 2AFC testing, the participant is presented with two stimuli, one of which is the target, and is forced to choose which is the correct stimulus. This contrasts with the tasks that we used previously (Sanders et al., 2017; Sanders & Jenkins, 2018), in which participants viewed individual stimuli, and made categorical judgements. There are several reasons why the proposed 2AFC testing should sharpen observers’ ability to distinguish hyper-realistic masks from real faces. First, the task instructions ensure that participants are aware in advance that masks will be presented. Second, social influence is minimised, as the task is computer based. Third, the task always involves two stimuli at a time: one is always a mask and the other is always a real face. Thus, even when participants are uncertain whether one of the images is the target, they can still solve the task indirectly if they are certain about the other image.

To test for other-race effects in this task, we collected data in both the UK and Japan. Although other-race effects are most strongly associated with identity-based tasks, such as face recognition (Meissner & Brigham, 2001) and face matching (Megreya, White, & Burton, 2011), our question here is whether they can also arise when distinguishing real faces from other face-like stimuli (Robertson et al., 2017) - a task more akin to face detection. The live viewing study by Sanders et al. (2017) could not address this point fully, as in naturalistic settings, the base-rate probabilities of encountering own-race and other-race faces are not well matched. Moreover, participants had no insight into the probability of a mask being present, even in the laboratory-based experiments. The 2AFC task gets around these limitations by allowing us to present own-race and other-race items equally often. We expect that equating background probabilities in this way will allow us to reach a more definitive answer.

## Experiment 1

To assess participants’ ability to distinguish hyper-realistic masks from real faces, we constructed a computer-based 2AFC task in which participants viewed pairs of on-screen images (one face and one mask), and indicated via key press which of the two images showed the mask. For comparison, we also included low-realism masks that were easy to detect. We expected that reaction times would be markedly slower in the *high-realism* condition than in the *low-realism* condition. Our main interest was whether the high-realism masks cleaved with the low-realism masks or with the real faces.

To test for other-race effects, we also presented equal numbers of own-race and other-race trials. The standard perceptual explanation of the other-race effect is that viewers become attuned to the variability that surrounds them, and remain relatively insensitive to variability outside of this range (e.g. O’Toole et al., 1994). These differences in perceptual experience lead to more efficient perceptual discrimination for own-race faces than for other-race faces. Although these effects are usually demonstrated using identification tasks, the same argument also applies to distinguishing hyper-realistic masks from real faces. We thus predicted shorter response latencies for own-race faces than for other-race faces in this task.

### Method

#### Ethics statement

Ethical approval for the experiment in this study was obtained from the departmental ethics committee at the University of York (approval number Id215) and Kyoto University (approval number 28-N-3). Participants provided written informed consent to participate.

#### Participants

Volunteers (*N* = 120) took part in exchange for a small payment or course credit. These were 60 members of the volunteer panel at the University of York (39 female, 21 male; mean age 23 years, age range 18–39 years) and 60 members of the volunteer panel at Kyoto University (27 female, 33 male; mean age 22 years, age range 18–50 years). Testing took place on site at Kyoto University, Japan and the University of York, UK.

### Materials and design

Three types of photographic image were used to construct the stimulus pairs - high-realism masks, low-realism masks, and real faces. To allow a fully crossed design, we collected an equal number of Asian and Caucasian images for each category. To ensure that we sampled real-world image variability, we used ambient images throughout (Jenkins et al., 2011). In the high-realism condition, a real face was paired with a hyper-realistic silicone mask. In the low-realism condition, a real face was paired with a non-realistic party mask.

#### High-realism mask images

To collect images of high-realism masks, we entered the search terms “realistic masks”, “hyper-realistic masks” and “realistic silicone masks” into Google Images. We selected images that (1) exceeded 150 pixels in height, (2) showed the mask in roughly frontal aspect, (3) showed the eye region without occlusions, and (4) included eyebrows made with real human hair. We used the same criteria to search the websites of mask manufacturers (e.g. RealFlesh Masks, SPFX, CFX) and topical forums on social media (e.g. Silicone Mask Sickos, Silicone Mask Addicts). For each of the Asian and Caucasian image sets, we gathered 37 hyper-realistic mask images that met the inclusion criteria (74 high-realism mask images in total).

#### Low-realism mask images

For comparison, we collected 74 images of low-realism masks by combining the search terms “Caucasian” and “Asian” with terms such as “Halloween”, “party”, “mask”, “masquerade”, “face-mask”, and “party mask” in Google Images, and selecting the first images that met the inclusion criteria 1–3 above. For low-realism mask images, race referred to the mask wearer, and was apparent from the parts of the face that were not occluded, and from the image source.

#### Real-face images

We also collected 148 real-face images to pair with the 74 high-realism and 74 low-realism mask images (148 mask images in total). To ensure that the demographic distribution among our real-face images was similar to that portrayed by the high-realism masks, we combined the search terms “Caucasian” and “Asian” with the terms “young male’”, “old male”, “young female”, and “old female” in Google Images. We then accepted images that met criteria 1–4 until the distribution of faces across these categories was the same as for the high-realism mask images. All photos were cropped to show the head region only and resized for presentation to 540 pixels high × 385 wide (see Fig. 2).

To create the stimulus displays, we paired each real-face image with a mask image from either the high-realism or the low-realism set. On each trial, the mask was equally likely to appear on the left or right side of the display. Stimuli always paired two images showing the same race (i.e. both Asian or both Caucasian). Within these constraints, image pairings were randomized separately for each participant, such that each participant saw each image exactly once, but judged different image combinations. In both the UK group and the Japan group, participants were randomly assigned to either the own-race or the other-race condition.

#### Procedure

Participants were instructed that each stimulus pair contained one real face and one mask, and that the task was to indicate via key press which image showed the mask. Each trial began with an image pair presented at the centre of the screen for 500 ms with the caption “Who is wearing the mask?” immediately below, and response options “Z” and “M” below the left and right images respectively (see Fig. 2). After 500 ms, the images were removed, and the question and response options remained onscreen until response. Participants pressed “Z” for the left image, or “M” for the right image as quickly and accurately as possible, and the response initiated the next trial. Each participant saw three practice trials followed by 74 recorded trials in a random order. The entire experiment took approximately 10 min to complete.

### Results

**Fig. 3:**
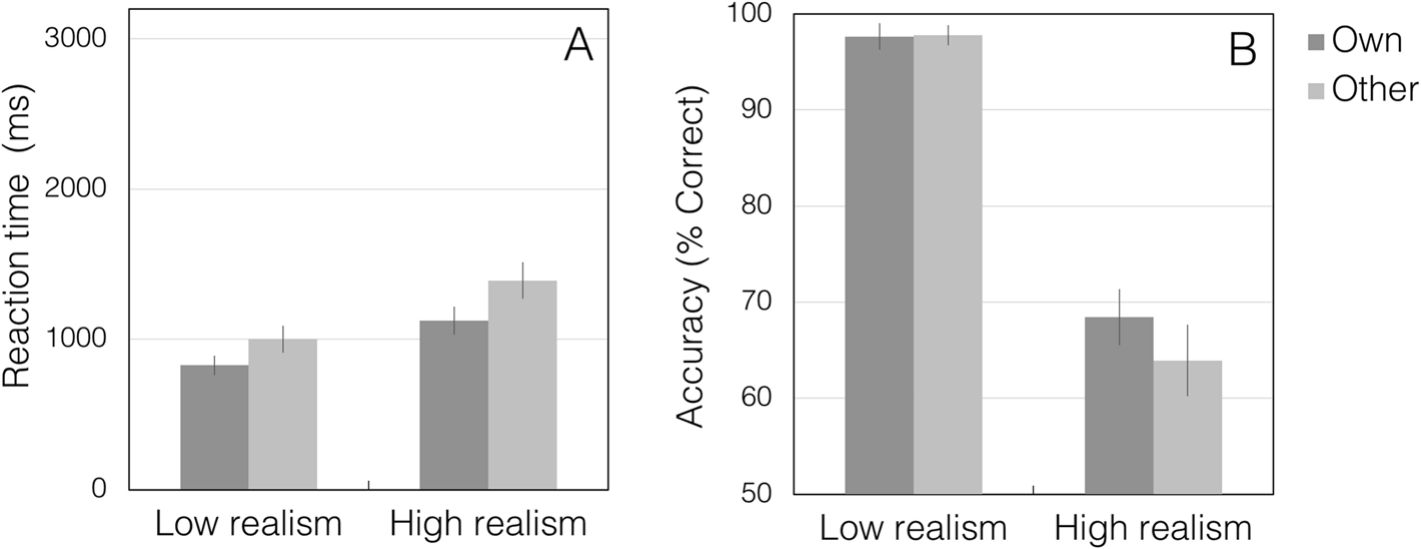
Reaction times (**a**) and percentage correct performance (**b**) in experiment 1. Error bars show 95% confidence intervals

Reaction time and error data are summarized in Fig. 3.

#### Reaction times

Participants’ mean correct reaction times (RTs) were analysed by 2 × 2 mixed analysis of variance (ANOVA) with the within-subjects factor of mask type (high-realism, low-realism), and the between-subjects factor of race (own-race, other-race).

As expected, there was a significant main effect of mask type, with slower responses for high-realism trials (mean (M) = 1258 ms, SE = 40.8, CI = 1178–1339) than for low-realism trials (M = 921 ms, SE = 29.3, CI 857–971) (*F* (1,118) = 204.6, *p* < .001, partial *η*^2^ = 0.63, Cohen’s *d* = 2.61).

There was also a significant main effect of race, with slower RTs in the other-race condition (M = 1197 ms, SE = 103.5, CI = 994–1399) than in the own-race condition (M = 976 ms, SE = 76.6, CI = 826–1125) (*F* (1,118) = 11.97, *p* < .001, partial *η*^2^ = 0.09, Cohen’s *d* = 0.63). The interaction between mask type and race was not significant (*F* (1,118) = 3.60, *p* = .06, partial *η*^2^ = 0.03, Cohen’s *d* = 0.35). For consistent reporting of effects across experiments, we also analysed simple main effects.

Simple main effects confirmed that there was a significant effect of mask type for both own-race (F (1,118 = 76.96, *p* < .001, partial *η*^2^ = 0.40, Cohen’s *d* = 1.63) and other-race faces (*F* (1,118 = 131.26, *p* < .001, partial *η*^2^ = 0.53, Cohen’s *d* = 2.12). The effect of race was also present in both the high-realism condition (*F* (1,118) = 11.62, *p* = .001; partial *η*^2^ = 0.09, Cohen’s *d* = 0.63) and the low-realism condition (*F* (1,118) = 9.61, *p* = .002; partial *η*^2^ = 0.08, Cohen’s d = 0.59).

#### Errors

Mean percentage correct scores were likewise analysed by 2 × 2 mixed ANOVA with the within-subjects factor of mask type (high-realism, low-realism), and the between-subjects factor of race (own-race, other-race).

This analysis revealed a significant main effect of mask type, with lower accuracy for high-realism trials (M = 66.2%, SE = 1.2, CI = 63.8–68.8) than for low-realism trials (M = 97.7%, SE = 0.4, CI = 96.9–98.6) (*F* (1,118) = 635.8, *p* < .001, partial *η*^2^ = 0.84, Cohen’s *d* = 4.58).

There was no main effect of race in errors (own-race: M = 83.0%, SE = 0.8, CI = 81.5–84.6; other-race: M = 80.9%, SE = 0.9, CI = 79.2–82.5) (*F* (1,118) = 2.69, *p* = .104, partial *η*^2^ = 0.02, Cohen’s *d* = 0.28), and no significant interaction between mask type and race (*F* (1,118) = 3.44, *p* = .066, partial *η*^2^ = 0.03, Cohen’s *d* = 0.35).

Simple main effects confirmed that there was a significant effect of mask type in both the own-race condition (*F* (1,118 = 272.85, *p* < .001, partial *η*^2^ = 0.70) and the other-race condition (*F* (1,118 = 366.33, *p* < .001, partial *η*^2^ = 0.76). Despite the numerical trend, there was no significant effect of race in the high-realism condition (*F* (1,118) = 3.45, *p* = .066, partial *η*^2^ = 0.03, Cohen’s *d* = 0.35), nor in the low-realism condition (*F* (1,118) = 0.02, *p* = .880, partial *η*^2^ < .001, Cohen’s *d* < .001).

Owing to the ceiling effect in the low-realism condition, we also compared own-race and other-race conditions with a separate Mann–Whitney test for each mask type. We found no significant effect of race for the high-realism condition (*U* = 1466, *p* = .079) or the low-realism condition (*U* = 1670, *p* = .437).

**Fig. 4:**
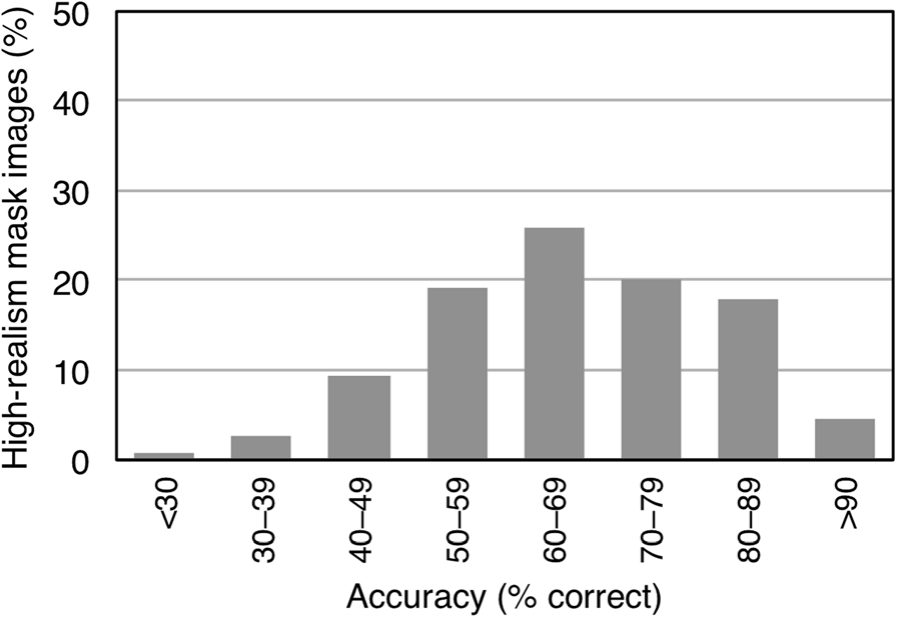
Distribution of responses for the high-realism mask images in experiment 1. The x-axis shows accuracy rates in 10% bins. The y-axis shows the proportion of images falling into each bin. Very few items fall into the highest accuracy bin

Given the high error rate in the high-realism condition, we next examined the distribution of errors across images. The purpose of this analysis was to establish whether errors were driven by a particular subset of images, or were instead distributed across the entire set. Figure 4 shows the results of this analysis. All of the high-realism mask images attracted some errors, and most attracted many errors. In other words, errors were not driven by a particular subset of images. Rather, they were distributed across the entire set.

### Discussion

Analysis of RTs showed that 2AFC discrimination of masks from real faces was indeed slower for high-realism masks than for low-realism masks (~ 300 ms RT cost). As it turned out, the more interesting effect was in the error data. Participants performed almost perfectly in the low-realism condition (98% accuracy). That is perhaps not surprising, given the simplicity of the task. However, accuracy in the high-realism condition was just 66%, in the context of chance performance being 50%. An error in this 2AFC task is striking, as it requires the observer to choose the real face over the alternative, *when the alternative is a mask*. The implication is not merely that the hyper-realistic masks looked human. In some cases, they appeared more human than human in this task. That was the judgement in one-third of the high-realism trials.

We also observed an effect of race in reaction times (~ 200 ms cost), though not in the accuracy data. If reliable, this is an intriguing finding, as it potentially extends the classic other-race effect from identification tasks to the very different task of differentiating real faces from synthetic faces (masks).

One aspect of our experiment that complicates interpretation is the limited exposure duration for the stimuli (500 ms). Limiting stimulus duration is standard practice when the task would otherwise be too easy (Bogacz et al., 2006). As it turned out, the high-realism condition was not too easy. In the next experiment, we removed this time limit.

## Experiment 2

In experiment 1, mask realism affected not only the speed of mask/face discriminations, but also their accuracy. One plausible interpretation of this result is that the hyper-realistic face masks were difficult to distinguish from real faces. However, another possibility is that the stimulus presentations were too brief (500 ms) to allow proper comparison of the two images. To distinguish these alternatives, we repeated the preceding experiment with one important change - stimuli now remained on screen until the participant responded. If errors in experiment 1 were due to insufficient viewing time, then unlimited viewing time should eliminate them. On the other hand, if the errors were due to the similarity of the masks to the faces, the error rate in the high-realism condition should remain high.

### Method

#### Participants

New volunteers (*N* = 120), none of whom participated in experiment 1, took part in exchange for a small payment or course credit. These were 60 members of the volunteer panel at the University of York (51 female, 9 male; mean age 20 years, age range 18–29 years) and 60 members of the volunteer panel at Kyoto University (23 female, 37 male; mean age 21 years, age range 18–38 years). Once again, testing took place on site at Kyoto University, Japan and the University of York, UK.

#### Materials and design

The images and experimental design were the same as in experiment 1, except that the stimulus pairs now remained on screen until the participant responded.

#### Procedure

The procedure was also the same as in experiment 1, except for the unlimited viewing time. Task instructions were modified to emphasize that the task was self-paced and that there was no time limit.

### Results

**Fig. 5:**
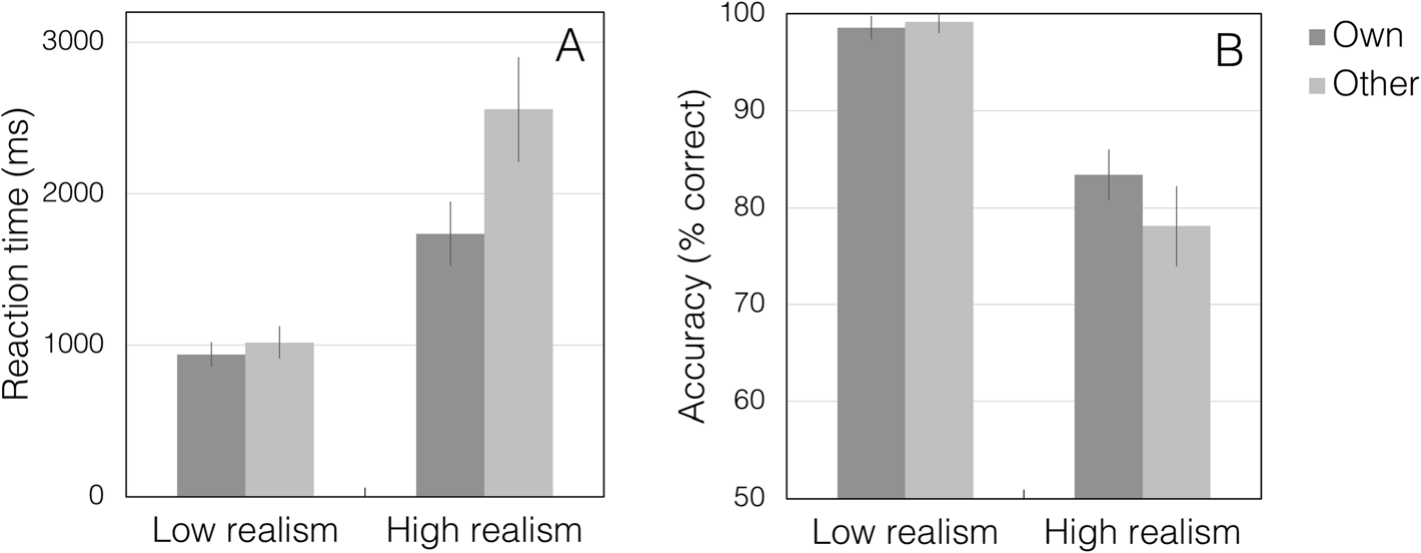
Reaction times (**a**) and percentage correct performance (**b**) in experiment 2. Error bars show 95% confidence intervals

Reaction time and error data are summarized in Fig. 5.

#### Reaction times

As in experiment 1, participants’ mean correct reaction times (RTs) were analysed by 2 × 2 mixed ANOVA with the within-subjects factor of mask type (high-realism, low-realism), and the between-subjects factor of race (own-race, other-race).

Once again, there was a large main effect of mask type, with slower responses for high-realism trials (M = 2146 ms, SE = 109.6, CI = 1931–2360) than for low-realism trials (M = 977 ms, SE = 33.9; CI = 911–1044) (*F* (1,118) = 213.2, *p* < .001, partial *η*^2^ = 0.64, Cohen’s *d* = 2.67).

There was also a significant main effect of race, with slower RTs overall for other-race trials (M = 1787 ms, SE = 219.8, CI = 1356–2217) compared with own-race trials (M = 1337 ms, SE = 142.9, CI = 1057–1617) (*F* (1,118) = 11.7, *p* < .001, partial *η*^2^ = 0.09, Cohen’s *d* = 0.63). On this occasion, there was a significant interaction between mask type and race (*F* (1,118) = 21.3, *p* < .001, partial *η*^2^ = 0.15, Cohen’s *d* = 0.84).

Simple main effects confirmed that there was a significant effect of mask type in both the own-race condition (*F* (1,118 = 49.86, *p* < .001, partial *η*^2^ = 0.30, Cohen’s *d* = 1.31) and the other-race condition (F (1,118 = 184.66, *p* < .001, partial *η*^2^ = 0.61, Cohen’s *d* = 2.50). The effect of race was driven specifically by the high-realism condition (*F* (1,118) = 15.70, *p* < .001, partial *η*^2^ = 0.12, Cohen’s *d* = 0.74), not the low-realism condition (*F* (1,118) = 1.40, *p* = .238, partial *η*^2^ = 0.01, Cohen’s *d* = 0.20).

#### Errors

Mean percentage correct scores were also analysed by 2 × 2 mixed ANOVA with the within-subjects factor of mask type (high-realism, low-realism), and the between-subjects factor of race (own-race, other-race).

Accuracy was again lower for high-realism trials (M = 80.8% correct; SE = 1.3; CI = 78.3–83.2) than for low-realism trials (M = 98.6%, SE = 0.42; CI = 98.0–99.7) (*F* (1,118) = 228.4, *p* < .001, partial *η*^2^ = 0.66, Cohen’s *d* = 2.79).

There was no overall main effect of race on accuracy (own-race: M = 88.6%, SE = 0.95, CI = 86.8–90.5; other-race: M = 91.0%, SE = 0.69, CI = 89.6–92.3) (*F* (1,118) = 2.73, *p* = .101, partial *η*^2^ = 0.02, Cohen’s *d* = 0.29). However, there was a significant interaction effect between mask type and race (*F* (1,118) = 6.08, *p* = .015; partial *η*^2^ = 0.49, Cohen’s *d* = 1.96).

Simple main effects confirmed that there was a significant effect of mask type in both the own-race condition (F (1,118 = 79.97, *p* < .001, partial *η*^2^ = 0.40) and the other-race condition (F (1,118 = 154.47, *p* < .001, partial *η*^2^ = 0.57). There was a significant effect of race in the high-realism condition (*F* (1,118) = 4.54, *p* = .035, partial *η*^2^ = 0.40, Cohen’s *d* = 1.63), but not in the low-realism condition (*F* (1,118) = .47, *p* = .495, partial *η*^2^ = 0.004, Cohen’s *d* = 0.13).

**Fig. 6:**
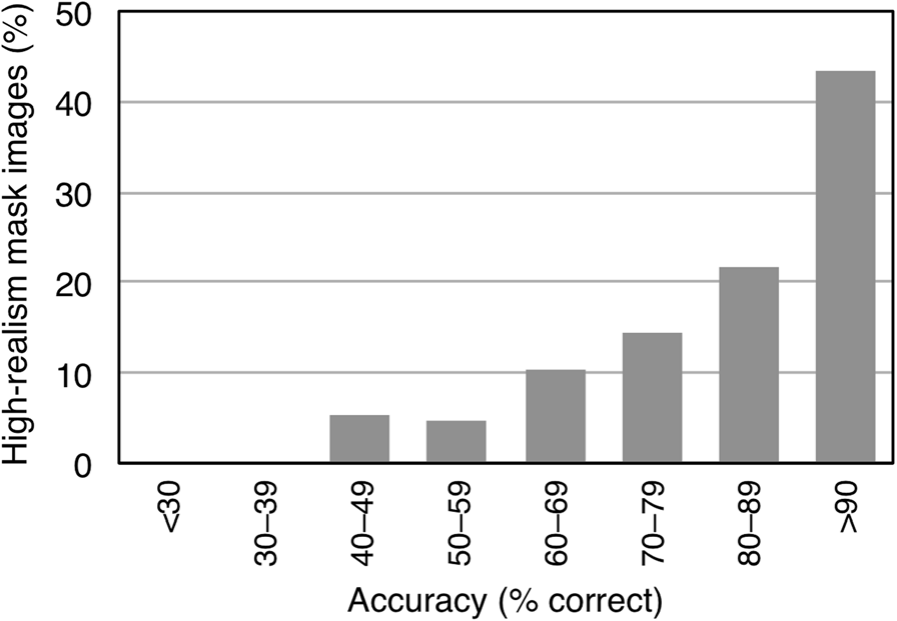
Distribution of responses for the High-realism masks images in Experiment 2. The x-axis shows accuracy rates in 10% bins. The y-axis shows the proportion of images falling into each bin

As in the preceding experiment, we also examined the distribution of errors across images (see Fig. 6). Consistent with the higher overall accuracy rate, the entire distribution was shifted higher in this experiment. Nevertheless, most of the high-realism mask images attracted some errors. Less than half of them were chosen with at least 90% accuracy.

### Discussion

Performance in the low-realism condition was virtually identical to experiment 1. Accuracy was again almost perfect (99%) in this easy task. Response times were also similar, despite the unlimited presentation time, suggesting that presentation time was not the limiting factor. In the high-realism condition, responses were much slower compared with the low-realism condition (~ 1100 ms cost), and compared with the high-realism condition in experiment 1 (~ 800 ms cost). Participants spent much longer on these difficult decisions, given the chance. However, even unlimited viewing time did not come close to eliminating errors. For one out of every five high-realism trials, participants judged the real face to be the mask.

As in experiment 1, there was also an effect of race in reaction times (~ 400 ms cost). This effect was carried mainly by the high-realism condition. This time however, the other-race cost in accuracy was also statistically significant - again, in the high-realism condition specifically (5% cost). Together, these measures indicate that distinguishing hyper-realistic masks from real faces was harder for other-race faces than for own-race faces.

## General discussion

To assess the realism of synthetic faces, specifically, hyper-realistic silicone masks, we tested how well viewers could distinguish photos of masks from photos of real faces in a 2AFC task. For low-realism masks, decisions were both fast and accurate. For high-realism masks, decisions were not only slower, but also surprisingly error prone. That was the finding in experiment 1, when viewing time was restricted (33% errors). It was also the finding in experiment 2, when viewing time was unlimited (20% errors). Whether making snap decisions (Gladwell, 2005) or more deliberative judgements (Kahneman, 2011), participants could not reliably distinguish hyper-realistic face masks from real faces.

It was already evident from real-world criminal cases (e.g. Henderson, 2016; Sabawi, 2018; Stanton, 2015), and from previous experimental work (Sanders et al., 2017), that hyper-realistic face masks can pass for real faces during live viewing. In principle however, other factors besides mask realism could account for those findings. For example, live viewing can place complex demands on attention, and challenging another person’s appearance may be socially awkward. The current studies reach similar conclusions based on comparison of photographs under laboratory conditions.

Although the error rates seen here are high, they almost certainly underestimate error rates that would arise in everyday settings. We chose the 2AFC task precisely because it works to the participant’s advantage. Participants knew from the outset that their task was mask detection, whereas in daily life that is not the default mindset. They also knew that every display contained a mask, whereas outside of the laboratory, the prevalence of hyper-realistic face masks is low (base rate is potentially important, as rare items are often missed; Wolfe, Horowitz, & Kenner, 2005). Finally, the mask in our displays was always one of two alternatives. The real world seldom presents the problem in such a convenient form. The more common task is to decide whether a single item is a mask or not (e.g. Sabawi, 2018; Stanton, 2015). Experimentally, viewers make many more errors in that task, even when they are briefed in advance about hyper-realistic face masks (Sanders & Jenkins, 2018); and many more again when they are not (Sanders et al., 2017).

None of this means that hyper-realistic mask detection is perceptually impossible. Accuracy in the current experiments was well above the chance level of 50%. However, in securing above-chance performance, we have retreated quite far from the applied problem. It is important not to lose sight of that retreat, because the applied problem presents many more difficulties.

Both experiments showed a clear cost for other-race comparisons relative to own-race comparisons. This cost emerged in reaction time measures (experiments 1 and 2) and also in error rates (experiment 2). Other-race effects have been shown repeatedly in the context of identification tasks. The present study demonstrates a similar effect in the very different context of discriminating real faces from synthetic faces. This aspect of our findings echoes two previous lines of work concerning classification of faces. Valentine (1991, experiment 5) asked participants to classify face images as intact or “jumbled” (features rearranged). Correct responses were slower for other-race faces than for own-race faces, consistent with greater distance from the norm in Valentine’s face space framework. The same account could explain the observed other-race effects in the current task. In more recent work on social groups, Hackel, Looser, and Van Bavel (2014) presented stimuli that were generated by morphing real faces with doll faces to create intermediate blends. Viewers perceived less humanness in a morphed face when it was assigned to an out-group than when it was assigned to an in-group, indicating out-group dehumanisation. The same phenomenon could account for the other-race effects seen here, if out-group dehumanisation blunts the distinction between real faces and hyper-realistic face masks. One way to test this possibility would be to assess mask/face discrimination for identical stimuli using a “minimal” group manipulation (Dunham, Baron, & Carey, 2011). Sanders et al. (2017) suggested that additional cues from unnatural movement or speech might improve mask detection in a live viewing task. To our knowledge, that has not yet been tested. However, given the present findings, it might be interesting to compare in-group and out-group appearance in that setting.

## Conclusion

We began by comparing the challenge of distinguishing synthetic faces from real faces to the Turing test. Our findings suggest that synthetic faces are at the point where they can fool viewers frequently. We see no reason to expect this imitation technology to stop improving. People are rightly wary of photorealistic images because they know that images can be manipulated. We may be entering a time where the same concerns apply to facial appearance in the real world.

## Supplementary information

**Additional file 1.** Licenses for images used in Fig. 2.

## Data Availability

Data and materials are available upon request.

## Abbreviations

2AFC: Two-alternative forced choice
ANOVA: Analysis of variance
RT: Reaction time
