# Supplementary material for: More human than human: a Turing test for photographed faces
Source: Cogn Res Princ Implic. 2019 Nov 21;4:43. doi: 10.1186/s41235-019-0197-9 (PMC6868074; doi:10.1186/s41235-019-0197-9)
Supplement: Supplementary file 1 — Additional file 1. Licenses for images used in Fig. 2. [file 41235_2019_197_MOESM1_ESM.docx]

| Trial | Z or Z | URL | Photographer/owner |
| --- | --- | --- | --- |
| 1 | Z | Taken by Author JGS | Jet G. Sanders |
| 1 | M | https://commons.wikimedia.org/wiki/File:Selfie_a_casa.jpg | Lluís Mestres Angla |
| 2 | Z | https://www.flickr.com/photos/practicalowl/33997210728 | Kit |
| 2 | M | https://www.freepik.com/free-photo/smiling-topless-woman-wearing-masquerade-carnival-mask-white-background_3735127.htm | Freepik |
| 3 | Z | http://bit.ly/2krCFvh | Flickr/Jan Krummrey |
| 3 | M | https://commons.wikimedia.org/wiki/File:Boyd_Holbrook_%2840061613100%29.jpg | Commons Wikimedia/ Greg2600 |
| 4 | Z | Taken by Author JGS | Jet G. Sanders |
| 4 | M | https://commons.wikimedia.org/wiki/File:Caroline_af_Ugglas_big_smile_2010.jpg | Commons Wikimedia/Bengt Nyman |
| 5 | Z | https://www.flickr.com/photos/sharman/8723083799 | Flickr/Martin Sharman |
| 5 | M | Taken by Author JGS | Jet G. Sanders |
